# Fermented soy supplementation improves indicators of quality of life: a randomized, placebo-controlled, double-blind trial in adults experiencing heartburn

**DOI:** 10.1186/s13104-020-05205-z

**Published:** 2020-08-03

**Authors:** Asmaa Fatani, Kadi Vaher, Daniela Rivero-Mendoza, Karima Alabasi, Wendy J. Dahl

**Affiliations:** 1 Department of Food Science and Human Nutrition, University of Florida, 359 FSHN Building, 572 Newell Drive, Gainesville, FL USA; 2 Lallemand Bio-Ingredients, Akadeemia tee 21/3, Tallinn, 12618 Estonia

**Keywords:** Gastroesophageal reflux, Soybean, *Lactobacillus*, Quality of life

## Abstract

**Objective:**

To determine if fermented soy supplementation relieves heartburn and improves gastrointestinal symptoms and quality of life, a randomized, double-blind parallel study was conducted with adults experiencing mild or moderate heartburn. Participants consumed up to 3, 1 g sachets of flavored, *Lactobacillus delbrueckii* fermented with soy flour (n = 23) or placebo (maltodextrin) (n = 27) sachets per heartburn incident as needed for 3 weeks. Symptom intensity at 5, 15, and 30 min post-administration was assessed using a Likert-like scale. The Gastrointestinal Symptoms Rating Scale (GSRS) and Gastro-esophageal Reflux Disease Quality of Life Questionnaire (GERD-QOL) were administered at baseline, post-intervention and following a 1-week washout.

**Results:**

No significant differences between groups were seen for heartburn severity or frequency, GSRS syndromes, or GERD-QOL domains. However, individual QOL items related to inconvenience of taking medications, fear of eating, inability to concentrate at work, and disturbance of after-meal activities and rest improved with fermented soy compared to placebo. Frequency of heartburn, diarrhea, and bloating improved during washout vs. baseline for the fermented soy group compared to placebo. *Lactobacillus delbrueckii* fermented soy supplementation improved QOL indicators and may decrease heartburn occurrence over time vs. an acute effect; efficacy of daily intake and longer duration requires investigation.

**Electronic supplementary material:**

The online version of this article (doi:10.1186/s13104-020-05205-z) contains supplementary material, which is available to authorized users.

## Introduction

Heartburn is common worldwide and prevalence may be increasing [1]. Symptoms are typically treated with medications such as antacids, proton pump inhibitors (PPI) and histamine-2 receptor antagonists. Although proton pump inhibitors are the standard of care for gastroesophageal reflux disease (GERD), these medications may not be effective for non-erosive disease [2]. Neither proton pump inhibitors nor histamine-2 receptor antagonists may be effective for intermittent use [3]. Further, antacids have been reported to cause side effects, including stomach pain, constipation, nausea and vomiting and must be used with caution by some patient populations such as those with kidney failure [4]. Thus, for occasional mild or moderate heartburn symptoms, efficacy of novel, non-pharmacological approaches to heartburn relief such as dietary supplements require exploration.

Fermentation of soy flour produces peptides which may have bioactive properties including modulation of inflammation [5]. However, no known published research has explored the efficacy of soy bioactive peptides on esophageal inflammation due to acid reflux and the associated symptoms. Further, the bacterial strain used for the fermentation process may also offer bioactivity and possibly, health benefit. *Lactobacillus delbrueckii* ssp. *delbrueckii* R-187, utilized in the commercial production of fermented soy, has been shown to modulate cytokine production in intestinal epithelial cells [6]. Clinical evidence suggests that fermented soy supplementation may lessen acid reflux [7]. The aim of this exploratory study was to evaluate the potential efficacy of *Lactobacillus delbrueckii* fermented soy supplementation on heartburn symptom relief, gastrointestinal symptoms, and heartburn-related quality of life. It was hypothesized that supplementation of fermented soy would reduce heartburn severity compared to placebo as well as reduce heartburn frequency and improve quality of life.

## Main text

### Methods

#### Study participants

Participants (18–60 years) were recruited between March and May 2019, from a university campus, by study staff using flyers and included if they experienced mild or moderate heartburn according to the Global Overall Symptom (GOS) questionnaire at least 2 days/week and used over-the-counter (OTC) products for heartburn, supplements, or dietary manipulation to relieve heartburn symptoms during the previous 3 months. Exclusion criteria included known allergy to soy, severe heartburn problem during the week prior to the study, previous or current treatment for any gastrointestinal diseases or illnesses, and being pregnant or lactating.

#### Study design and procedures

A 5-week, randomized, double-blind, placebo-controlled, parallel-group study was conducted in Florida, US. Following informed consent, weight (Seca^®^ 874 flat scale) and height (Seca^®^ 217 portable stadiometer) were measured and demographic information was collected. Participants completed a 1-week baseline and on day 8, after confirmation of the inclusion/exclusion criteria, were randomized to receive either fermented soy or placebo (maltodextrin) for 3 weeks, followed by a 1-week washout. A 3-week supply of orange-flavored supplements was provided in coded, identical packaging (9, 1 g opaque sachets/day). The fermented soy supplement was produced by the fermentation of toasted soybean flour by *Lactobacillus delbrueckii* ssp. *delbrueckii* R-187 and subsequently heat-treated (Lallemand Bio-Ingredients Inc., Grenaa, Denmark). The placebo was maltodextrin (Archer Daniels Midland Company, Tianjin, China) and flavoring. Randomization, using a sealed envelope method, was stratified by symptom severity (mild and moderate) and frequency (rare and frequent), and was completed by an individual not otherwise involved in the study. Participants recorded the consumption of study supplements and any OTC medication in booklets provided. Participants were asked to maintain their dietary habits during the study, with the exception of discontinuing intake of protein supplements, protein bars and/or protein shakes prepared with protein powders (concentrates or isolates) as well as tofu.

Participants completed daily questionnaires regarding heartburn severity (primary outcome) and frequency (secondary outcome) using a Likert-like scale (1 = no symptoms to 5 = severe discomfort) and noted any OTC heartburn medication use throughout the study. Participants were instructed to consume 1 sachet upon heartburn event and then record symptom intensity at 5, 15 and 30 min after administration. If the supplement did not alleviate the heartburn symptoms completely, the participant could take another sachet and repeat the symptom reporting. If the heartburn symptoms persisted beyond the second 30-min period, participants could take a third supplement or follow their regular routine with heartburn incidence (including administration of an OTC heartburn medication). Unused sachets were returned post-intervention. Compliance to supplementation was determined by supplement intake as a percentage of heartburn events. Adverse events were queried and other secondary outcomes, including the Gastrointestinal Symptom Rating Scale (GSRS) (1-no discomfort to 7-very severe discomfort) [8] and Gastro-oesophageal Reflux Disease Quality of Life Questionnaire (GERD-QOL) (4-strongly disagree to 0-strongly agree) [9], were assessed during study visits at the end of each period. In addition, three, 24-h dietary recalls per period were collected and analyzed using the Automated Self-Administered 24-h (ASA24) Dietary Assessment Tool, version (2018), developed by the National Cancer Institute, Bethesda, MD as a compliance measure.

#### Statistical analysis

Sample size was based on achieving a clinically meaningful effect of reducing heartburn severity to 2 (slight discomfort) on the severity scale of 1 (no symptoms) to 5 (severe discomfort). Given a mean severity of 2.6 ± 0.7 (alpha, 0.05; power, 80%), a sample size of 42 (21 per group) was needed. A sample size of 50 was targeted to accommodate potential dropouts. The Wilcoxon Signed Rank was used to test the differences between groups at baseline and to test for differences in the change from baseline within each arm for severity by time, heartburn frequency, and GSRS syndromes. A linear mixed model was used to test for differences in the GERD-QOL domains over the three time periods, baseline, intervention, and washout; and between study groups. Individuals were treated as random effects, with an unstructured co-variance to account for the repeated measures and non-homogeneity of variances. Fixed effects in the model were interventions, periods, and their interaction. Alpha was set at 0.05.

### Results

**Fig. 1:**
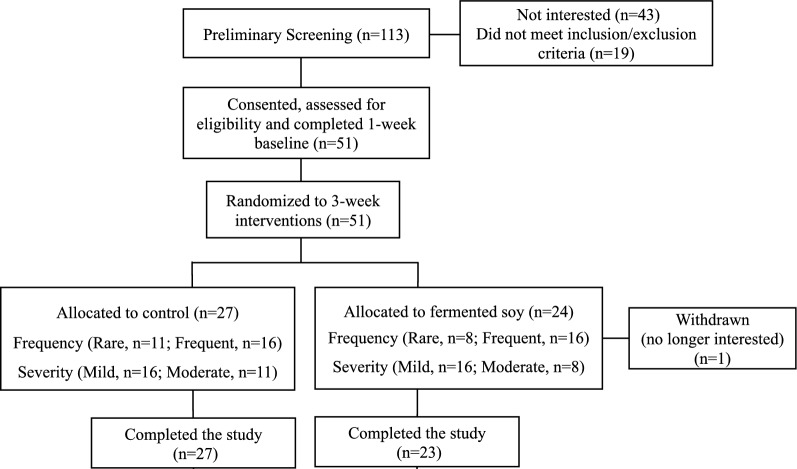
Participant recruitment, randomization and study flow

Participant recruitment, randomization and study flow are shown in Fig. 1. The demographic characteristics of the study participants and compliance are presented in Additional file 1: Table S1. Intake of study supplements during the intervention period was 15.3 ± 18.3 and 15.0 ± 11.2 sachets/participant for fermented soy and placebo group, respectively. No differences were seen for OTC medication intake (fermented soy: 0.4 ± 0.9; placebo: 0.6 ± 1.4 times/person). For both the fermented soy and placebo groups, 59% of the participants reported that they believed they were receiving the fermented soy. Although participants were instructed to avoid food sources of isolated protein throughout the study, 3 and 5 participants, in the fermented soy and placebo, respectively, reported intake of such foods. No study-related adverse events were reported.

**Fig. 2:**
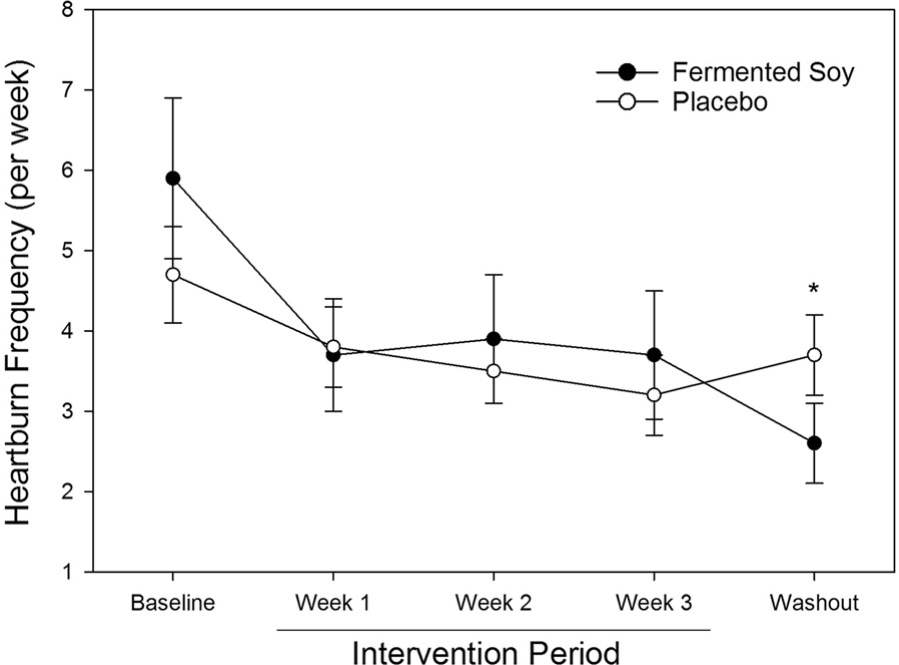
Heartburn frequency by week reported by participants experiencing mild to moderate heartburn randomized to fermented soy (n = 23) and placebo (n = 27). * p < 0.05

Reported heartburn severity did not differ between groups during any periods. During the intervention period, differences from T0 to T5 (soy: − 0.4 ± 0.6; placebo: − 0.4 ± 0.6), T0 to T15 (soy: − 0.6 ± 0.7; placebo: − 0.7 ± 0.8) and T0 to T30 (soy: − 0.9 ± 0.9; placebo: − 1.1 ± 0.9) did not differ (NS). Heartburn frequency by week is shown in Fig. 2. No significant difference was seen for heartburn frequency from baseline between groups during the intervention period; however, fermented soy significantly reduced heartburn frequency compared to the placebo during the washout period. Regarding GSRS syndromes, changes from baseline to intervention and baseline to washout values did not significantly differ between fermented soy and placebo (Additional file 2: Table S2). However, there were significant differences in the delta (washout minus baseline) between the soy and placebo groups, for the individual GSRS items, “Have you been bothered by diarrhea during the past week?” (soy: 0.3 ± 1.4 *vs* placebo: − 0.3 ± 1.2, p < 0.05) and “Has your stomach felt bloated during the past week?” (soy: 0.7 ± 1.7 vs placebo: 0.1 ± 1.3, p < 0.05), representing an improvement in the fermented soy group.

**Fig. 3:**
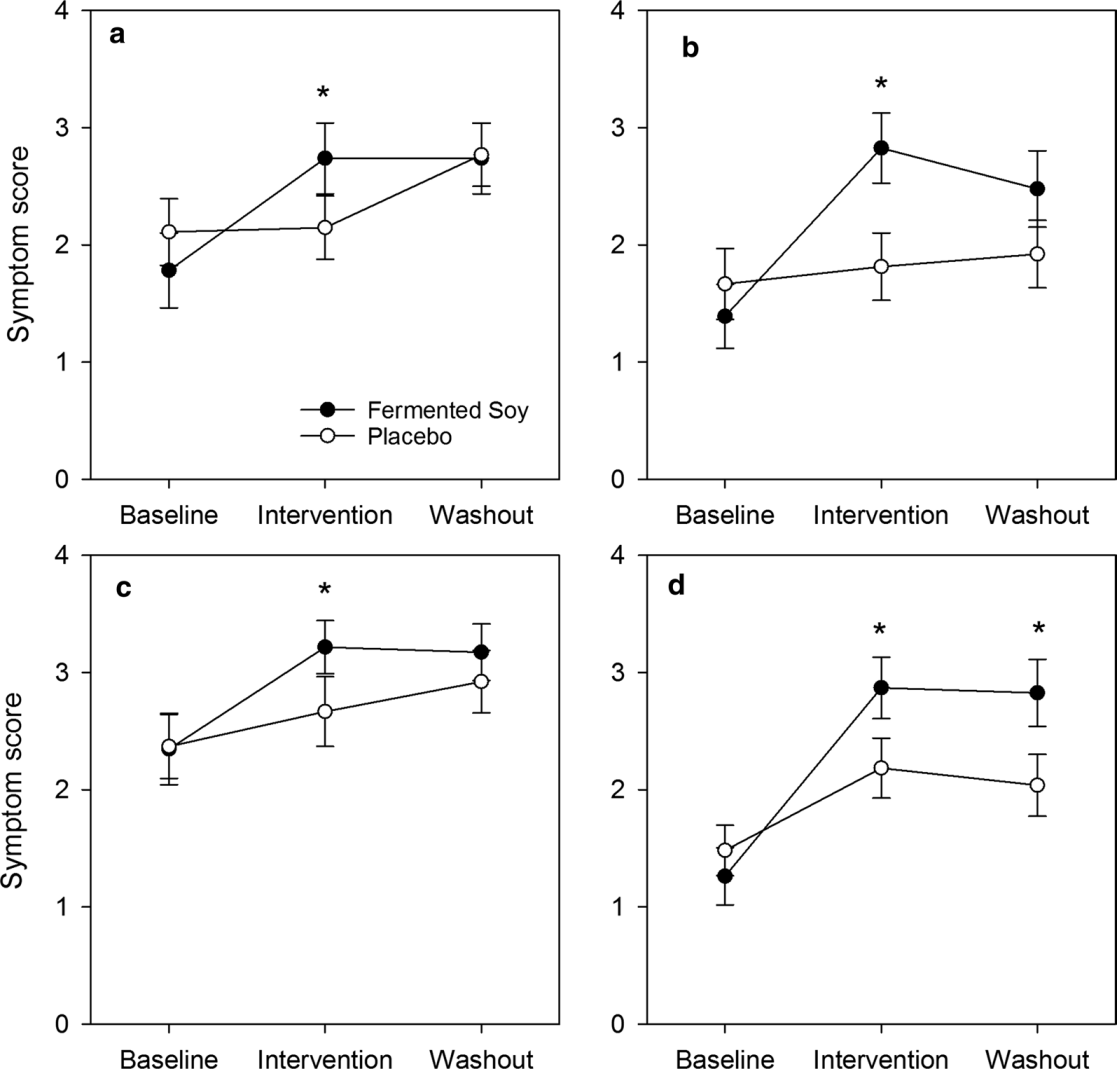
Gastro-oesophageal Reflux Disease Quality of Life Questionnaire (GERD-QOL) items reported by period in individuals experiencing mild or moderate heartburn randomized to fermented soy (n = 23) and placebo (n = 27). * p < 0.05. Scale: 0—Strongly Agree to 4—Strongly Disagree. Reponses to: **a** “I found it inconvenient to have to take medications regularly because of acid reflux and heartburn symptoms” **b** “I was afraid to eat too much because of acid reflux and heartburn symptoms” **c** “I was unable to concentrate on my work because of acid reflux and heartburn symptoms” **d** “Acid reflux and heartburn symptoms disturbed my after-meal activities or rest” [9]

The responses to the GERD-QOL domains are presented in Additional file 2: Table S2; there were no significant differences between the intervention groups. However, as shown in Fig. 3, during intervention vs. baseline there were significant differences favoring fermented soy for the responses to the GERD-QOL items including: “I found it inconvenient to have to take medications regularly because of acid reflux and heartburn symptoms” (− 1.0 ± 1.3 vs − 0.04 ± 1.8, p < 0.05), “I was afraid to eat too much because of acid reflux and heartburn symptoms,” (− 1.4 ± 1.3 vs − 0.2 ± 1.7, p < 0.05), “I was unable to concentrate on my work because of acid reflux and heartburn symptoms” (− 0.9 ± 1.6 vs − 0.3 ± 1.0, p < 0.05), and “Acid reflux and heartburn symptoms disturbed my after-meal activities or rest” (− 1.6 ± 1.5 vs − 0.7 ± 1.5, p < 0.05). There were also significant differences from baseline favoring fermented soy between baseline and washout for “fear of eating favorite foods or drinks” (− 1.3 ± 1.6 vs − 0.4 ± 1.5, p < 0.05) and “disturbance of after-meal activities and rest” (− 1.6 ± 1.7 vs − 0.5 ± 1.6, p < 0.05).

### Discussion

We sought to determine the efficacy of fermented soy supplementation on heartburn relief, gastrointestinal symptoms, and heartburn-related quality of life. In general, the fermented soy, when administered per heartburn event, alleviated heartburn severity by time and frequency similarly to the placebo. However, those participants receiving fermented soy showed a reduction in heartburn frequency during washout and a downwards trend from baseline (from 5.9 to < 3 heartburn events per week), suggesting that there may be an effect of the fermented soy supplementation over time. It is possible that, prophylactic administration, perhaps daily, may decrease heartburn incidents over time through modulation of inflammation. Research is needed to determine if soy bioactive peptides and/or the inactivated *L. delbrueckii* ssp. *delbrueckii* R-187 mitigate esophageal inflammation and thereby, heartburn symptoms. *L. delbrueckii* ssp. *delbrueckii* R-187 has previously been shown to significantly down-regulate the production of interleukin-8 and tumor necrosis factor-α in intestinal cells culture [6]. It has been shown that pro-inflammatory cytokine production is increased before tissue destruction and precedes the loss of mucosal integrity [10]. Consequently, by reducing pro-inflammatory cytokines, a decrease of inflammation of the epithelial cells may occur over time. Studies are needed to confirm this hypothesis.

Fermented soy did not significantly improve gastrointestinal syndromes or quality of life domains compared to placebo. Nevertheless, individual quality of life items improved with the fermented soy. Previous research suggests a benefit from heartburn symptom relief on quality of life. Jiang et al. showed that responders to heartburn treatment achieved superior improvement of quality of life compared with non-responders [11]. In the present study, although a reduction of heartburn frequency was observed only during washout period and not during the intervention period, enhancements, of some aspects of quality of life, specifically less fear of eating and disruptions of work and activities, were reported during the supplementation period with fermented soy, preceding the mitigation.

Fermented soy supplementation was feasible given the high rate of adherence and participant retention. In individuals with mild to moderate heartburn, fermented soy improved some indicators of heartburn-related quality of life and may have potential benefits for reducing heartburn frequency over time. Future research should examine whether reduction in heartburn frequency occurs with daily prophylactic supplementation over time.

## Limitations

The study has limitations worth noting. The placebo demonstrated similar efficacy for the suppression of heartburn severity by time as the fermented soy supplement, suggesting a strong placebo effect or possibly, but unlikely, a symptom-suppressing effect of the placebo (orange-flavored maltodextrin). The placebo effect was also seen in the GSRS reflux syndrome as well as the heartburn-related quality of life domains. A placebo effect is supported by the findings that a similar percentage of participants in both groups (59%) believed that they were receiving the fermented soy. The significant placebo effect was unexpected and therefore, not taken into consideration with the sample size determination. As this was the first study investigating the efficacy of fermented soy on heartburn severity in adults experiencing mild and moderate heartburn, the trial was designed to test an acute effect of supplementation in accordance with how similarly formulated, commercial supplements are indicated. Instead, the trending decrease in heartburn frequency suggests a beneficial health effect over time, which supports a potential inflammation-modulating hypothesis. The present study may not have had adequate dosing or length to demonstrate a significant effect on heartburn events during the intervention period, particularly given the substantial placebo effect.

## Supplementary information

**Additional file 1: Table S1.** Characteristics of study participants and compliance.

**Additional file 2: Table S2.** Gastrointestinal Symptom Response Scale (GSRS) syndrome scores and Gastro-oesophageal Reflux Disease Quality of Life Questionnaire (GERD-QOL) domains reported per period by adults with mild to moderate heartburn symptoms receiving fermented soy *vs*. placebo.

## Data Availability

The datasets generated and/or analyzed during the current study are not publicly available due to human subject confidentiality but deidentified data are available from the corresponding author upon reasonable request.

## Abbreviations

ASA24: Automated Self-Administered 24-hour
GERD: Gastroesophageal Reflux Disease
GERD-QOL: Gastro-esophageal Reflux Disease Quality of Life Questionnaire
GOS: Global Overall Symptom
GSRS: Gastrointestinal Symptoms Rating Scale
OTC: over-the-counter
