# Supplementary material for: Fermented soy supplementation improves indicators of quality of life: a randomized, placebo-controlled, double-blind trial in adults experiencing heartburn
Source: BMC Res Notes. 2020 Aug 3;13:364. doi: 10.1186/s13104-020-05205-z (PMC7397630; doi:10.1186/s13104-020-05205-z)
Supplement: Supplementary file 1 — Additional file 1: Table S1. Characteristics of study participants and compliance. [file 13104_2020_5205_MOESM1_ESM.docx]

Table S1. Characteristics of study participants and compliance.

|  | Fermented Soy | Placebo |
| --- | --- | --- |
| Gender, male/female, n | 8/16 | 6/21 |
| Age, years, median (range) | 30 (18-55) | 24 (19-56) |
| Race, n (%) |  |  |
| Asian | 5 (21) | 3 (11) |
| Black | 3 (13) | 6 (22) |
| White | 14 (58) | 14 (52) |
| More than One Race | 0 (0) | 1 (4) |
| Other (Middle East) | 2 (8) | 3 (11) |
| Ethnicity, n (%) |  |  |
| Hispanic | 3 (13) | 3 (11) |
| Non-Hispanic | 21 (87) | 23 (85) |
| Unknown/Not reported | 0 | 1 (4) |
| Body Mass Index (BMI) |  |  |
| Normal (18.5 to 24.9) | 13 | 13 |
| Overweight (25 to 29.9) | 5 | 8 |
| Obese (over 30) | 6 | 6 |
| Dietary intake at baseline |  |  |
| Energy (kcal) | 1618±115 | 1704±135 |
| Protein (g) | 71.7±8.8 | 74.9±7.9 |
| Total Fat (g) | 67.4±6.1 | 67.6±6.2 |
| Carbohydrate (g) | 185.2±15.9 | 201.0±20.7 |
| Fiber (g) | 15.3±1.6 | 14.6±1.6 |
| Heartburn severity at baseline |  |  |
| Mild (Global Overall Symptom, 2-3) | 16 (67) | 16 (59) |
| Moderate (Global Overall Symptom, 4-5) | 8 (33) | 11 (41) |
| Heartburn frequency at baseline |  |  |
| Rare (2-3 heartburn incidents/week) | 8 (33) | 11 (41) |
| Frequent (4 ≤ heartburn incidents/week) | 16 (67) | 16 (59) |
| Compliance to supplement protocol | 92% | 91% |
