# Supplementary material for: Fermented soy supplementation improves indicators of quality of life: a randomized, placebo-controlled, double-blind trial in adults experiencing heartburn
Source: BMC Res Notes. 2020 Aug 3;13:364. doi: 10.1186/s13104-020-05205-z (PMC7397630; doi:10.1186/s13104-020-05205-z)
Supplement: Supplementary file 2 — Additional file 2: Table S2. Gastrointestinal Symptom Response Scale (GSRS) syndrome scores and Gastro-oesophageal Reflux Disease Quality of Life Questionnaire (GERD-QOL) domains reported per period by adults with mild to moderate heartburn symptoms receiving fermented soy vs. placebo. [file 13104_2020_5205_MOESM2_ESM.docx]

Table S2. Gastrointestinal Symptom Response Scale (GSRS) syndrome scores and Gastro-oesophageal Reflux Disease Quality of Life Questionnaire (GERD-QOL) domains reported per period by adults with mild to moderate heartburn symptoms receiving fermented soy *vs*. placebo.

|  | GSRS Syndromes | Fermented Soy | Placebo | GERD-QOL Domains | Fermented Soy | Placebo |
| --- | --- | --- | --- | --- | --- | --- |
|  |  | mean ± SE | |  | mean ± SE | |
| Baseline | Abdominal pain | 2.2±0.2 | 2.2±0.1 | Daily Activity | 2.4 ± 0.2 | 2.3 ± 0.2 |
| Intervention |  | 1.9±0.2 | 2.0±0.2 |  | 3.1 ± 0.2 | 2.6 ± 0.2 |
| Washout |  | 1.8±0.2 | 2.0±0.2 |  | 3.1 ± 0.2 | 2.8 ± 0.2 |
| Baseline | Reflux | 3.0±0.2 | 3.1±0.2 | Diet | 1.9 ± 0.2 | 1.8 ± 0.2 |
| Intervention |  | 2.8±0.2 | 2.7±0.2 |  | 2.9 ± 0.2 | 2.2 ± 0.2 |
| Washout |  | 2.2±0.2 | 2.5±0.2 |  | 2.8 ± 0.2 | 2.2 ± 0.2 |
| Baseline | Indigestion | 2.3±0.2 | 2.5±0.2 | Psychological Well-being | 2.3 ± 0.2 | 2.2 ± 0.2 |
| Intervention |  | 1.8±0.2 | 2.2±0.2 |  | 2.7 ± 0.2 | 2.6 ± 0.2 |
| Washout |  | 1.7±0.2 | 2.1±0.2 |  | 3.0 ± 0.3 | 2.6 ± 0.2 |
| Baseline | Diarrhea | 1.8±0.2 | 1.5±0.1 | Treatment Effect | 2.5 ± 0.3 | 2.3 ± 0.3 |
| Intervention |  | 1.6±0.2 | 1.4±0.1 |  | 3.0 ± 0.2 | 2.4 ± 0.2 |
| Washout |  | 1.5±0.2 | 1.5±0.2 |  | 3.1 ± 0.2 | 2.7 ± 0.3 |
| Baseline | Constipation | 1.7±0.2 | 1.7±0.2 |  |  |  |
| Intervention |  | 1.4±0.1 | 1.4±0.1 |  |  |  |
| Washout |  | 1.6±0.2 | 1.5±0.1 |  |  |  |

GSRS: Gastrointestinal Symptom Response Scale. GERD-QOL: Gastro-oesophageal Reflux Disease Quality of Life Questionnaire. No significant differences.
